# What do you mean by engagement? – evaluating the use of community engagement in the design and implementation of chronic disease-based interventions for Indigenous populations – scoping review

**DOI:** 10.1186/s12939-020-01346-6

**Published:** 2021-01-06

**Authors:** Sahr Wali, Stefan Superina, Angela Mashford-Pringle, Heather Ross, Joseph A. Cafazzo

**Affiliations:** 1 Institute of Health Policy, Management and Evaluation, Dalla Lana School of Public Health, University of Toronto, Toronto, ON Canada; 2 Centre for Global eHealth Innovation, Toronto General Hospital, Techna Institute, University Health Network, R. Fraser Elliott Building, 4th floor, 190 Elizabeth St, Toronto, ON M5G 2C4 Canada; 3 Waakebiness-​Bryce Institute for Indigenous Health, Dalla Lana School of Public Health, University of Toronto, Toronto, ON Canada; 4 Translational Research, Department of Laboratory Medicine and Pathobiology, Faculty of Medicine, University of Toronto, Toronto, ON Canada; 5 Ted Rogers Centre for Heart Research, University Health Network, Toronto, ON Canada; 6 Faculty of Medicine, Institute of Medical Sciences, University of Toronto, Toronto, ON Canada; 7 Peter Munk Cardiac Centre, University Health Network, Toronto, ON Canada; 8 Institute of Biomaterials and Biomedical Engineering, University of Toronto, Toronto, ON Canada

**Keywords:** Indigenous, Community engagement, Chronic disease, Participatory research

## Abstract

**Background:**

Indigenous populations have remained strong and resilient in maintaining their unique culture and values, despite centuries of colonial oppression. Unfortunately, a consequential result of facing years of adversity has led Indigenous populations to experience a disproportionate level of poorer health outcomes compared to non-Indigenous populations. Specifically, the rate of Indigenous chronic disease prevalence has significantly increased in the last decade. Many of the unique issues Indigenous populations experience are deeply rooted in their colonial history and the intergenerational traumas that has subsequently impacted their physical, mental, emotional and spiritual well-being. With this, to better improve Indigenous health outcomes, understanding the local context of their challenges is key. Studies have begun to use modes of community engagement to initiate Indigenous partnerships and design chronic disease-based interventions. However, with the lack of a methodological guideline regarding the appropriate level of community engagement to be used, there is concern that many interventions will continue to fall short in meeting community needs.

**Objective:**

The objective of this study was to investigate the how various community engagement strategies have been used to design and/or implement interventions for Indigenous populations with chronic disease.

**Methods:**

A scoping review guided by the methods outlined by Arksey and O’Malley was conducted. A comprehensive search was completed by two reviewers in five electronic databases using keywords related to community engagement, Indigenous health and chronic disease. Studies were reviewed using a descriptive-analytical narrative method and data was categorized into thematic groups reflective of the main findings.

**Results:**

We identified 23 articles that met the criteria for this scoping review. The majority of the studies included the use a participatory research model and the procurement of study approval. However, despite the claimed use of participatory research methods, only 6 studies had involved community members to identify the area of priority and only five had utilized Indigenous interview styles to promote meaningful feedback. Adapting for the local cultural context and the inclusion of community outreach were identified as the key themes from this review.

**Conclusion:**

Many studies have begun to adopt community engagement strategies to better meet the needs of Indigenous Peoples. With the lack of a clear guideline to approach Indigenous-based participatory research, we recommend that researchers focus on 1) building partnerships, 2) obtaining study approval and 3) adapting interventions to the local context.

## Introduction

Despite facing centuries of colonization and cultural genocide, Indigenous populations have remained resilient in maintaining their cultural identity through their connection with their values and the land [1]. Nevertheless, even with the strengths of the Indigenous Peoples, according to the International Indicators for Health, Indigenous health status has been reported to be among the lowest in the world [1]. Specifically, while the rate of communicable disease has declined in most Indigenous populations, the rise in chronic disease prevalence has significantly increased [1, 2]. This disproportionate level of poorer health outcomes experienced by Indigenous People compared to non-Indigenous People, can be attributed due to a series of complex factors including the disparities in the social determinants of health (SDH) (e.g. housing, income, food security, racism, discrimination, access to care services, climate change) and the deeply rooted impacts of colonialism (i.e. historical trauma) [2, 3].

With the increasing recognition for various SDH influencing health outcomes, strategies to support Indigenous health have begun to shift in reflection of these highlighted needs [4]. Traditionally, Indigenous-focused research has utilized Western-based methods to investigate modes to improve Indigenous health status. These studies have often had remarkably little success in achieving their desired outcomes, mainly due to the investigators a priori assumption of the issue to be addressed and the intervention to resolve it [4–6]. It is also important to note that in many cases, health system data regarding Indigenous health may not even be representative of the community-specific conditions, as it has become common for data only reflecting the state of a subset of an Indigenous population to become widely disseminated as a general statistic [7]. Each Indigenous population faces unique challenges that may not be applicable to others, but this contextual significance is often lost within large national datasets, leaving chronic issues of priority to be overlooked [7, 8]. With this, various Indigenous governing bodies have called on researchers to recognize the importance of context and to increase partnership efforts to better assist community-specific care priorities [1, 7]. Thus, with this growing recognition for the SDH and the call to action for collaboration, this shift in Indigenous-based research has involved the integration of numerous community engagement strategies within general research practice.

One term that has become more commonly used to define the suite of community engagement approaches used in Indigenous populations, is participatory research (PR) [4–6]. PR is a type of study design that places community members at the center of the research process in order to empower Indigenous voices, strengthen community capacity and promote meaningful change [5, 6]. Unlike other research designs that work from the top down, PR utilizes a grassroots collaborative approach to build genuine, equitable partnerships between researchers and communities [5, 6]. Within PR, there are an array of different research approaches, all of which differ in their methods, goals and degree of participation. This includes but is not limited to the following study designs; participatory action research (PAR), community-based participatory research (CPBR), and community research (CR) [9, 10]. PAR is characterized by a cycle of reflective inquiry undertaken by communities and researchers to create action for positive social change [11, 12]. True PAR allows the community to lead all stages of the research from identifying the issue of concern to the resultant mode of action, in addition to following the First Nations principles of OCAP (Ownership, Control, Access, Possession) and Inuit Qaujimajatuqangit (IQ) [12]. Similarly, CBPR is guided by principles that call for equitable relations and co-learning among researchers and communities to integrate both Western and traditional Indigenous knowledges. Active community engagement in all stages of the research process is encouraged, but the key goal is to develop interventions that are beneficial, sustainable, and locally relevant [12]. CR follows the same principles as PAR and CPBR, however there are less defined guidelines on its research procedures. Ultimately, the foundational premise of all PR approaches involves the need to work with communities to understand the underlying factors responsible for their health outcomes [5, 6].

Despite the ideologies behind these various PR approaches, studies indicating the use of PAR or CPBR have included varying features within their methods that do not explicitly align with one another’s study design classifications [12]. The lack of an agreed definition regarding these terms, has created a sense of ambiguity with respect to the appropriate level of engagement to be used. Many studies have specified that they have engaged with communities or conducted PR, but there is a lack of clarity regarding what the term ‘engagement’ entails for each researcher, with respect to their study methods and principles. Instead, terms of community engagement or PR are often used in a tokenistic manner, without properly evaluating the methodological validity of its claimed use. For example, a study evaluating the impact of a health education program for diabetes, stated their research had followed PR guidelines, yet within their methods, the study did not even consult the Indigenous students the program was designed for [13]. Within the literature, many studies on Indigenous-based research have focused on understanding the appropriate modes to engage with Indigenous communities, but have failed to provide a clear guide on how these approaches should be integrated within the research process to develop meaningful interventions [10, 12]. With the lack of a proper definition or methodological guideline regarding PR, there is a need to evaluate the varying modes of PR or community engagement used to better guide future studies on Indigenous health. To address this gap, the aim of this review is to explore the level of community engagement (i.e. inclusion of community consult across different stages of research process) used for the design and/or implementation of chronic disease-based interventions for Indigenous populations.

## Methods

### Study design

A systematic scoping review guided by the methodological framework developed by Arksey and O’Malley was conducted [14]. The steps of this framework utilize an iterative review process over the course of five phases: 1) identify research question, 2) identify relevant studies, 3) study selection, 4) charting the data and 5) summarizing and reporting the results [14]. This review approach is particularly relevant as it helps to map key findings from the literature to better understand the varying levels of engagement used when developing interventions for Indigenous communities [14, 15].

### Research question

The focus of this review was to explore the use of community engagement to design and/or implement interventions for Indigenous populations with chronic disease. While the chronic disease-related health disparities among Indigenous People are undeniable, it is unknown whether, and to what extent, interventions have been designed with the unique community perspective. Thus, this led to the following guiding research question:

*What is known in the literature on the level of community engagement or participatory research used for the design and/or implementation of chronic disease-related interventions for Indigenous populations?*

### Search strategy

A preliminary scan of literature was conducted in two academic databases (Medline and the Bibliography of Native North Americans) with the following search terms: community engagement, Indigenous and chronic disease. With support from a faculty-affiliated librarian, keywords and related subject headings were refined according to text contained in the title and abstract of the initial literature search (Table 1). Based on the keywords identified, one reviewer conducted a comprehensive search in five electronic databases: Medline, Bibliography of Native North Americans, Embase CINAHL and Scopus. Reference lists of relevant articles were also reviewed to extract additional studies that were not found in the initial search. This review did not restrict studies according to the year of publication or the country of origin. However, studies were excluded if they were not published in English or if they were grey literature, review papers or study protocols.

**Table 1:** Scoping Review Search Strategy

**Scoping Review Keywords**	
1. Community engagement	
• Community engagement OR participatory research OR community based participatory research OR participatory action research OR community institutional relation* OR community participation OR action research OR participatory research OR participatory engagement OR participation OR community research	
**AND**	
2. Indigenous	
• Indigenous OR Indigenous People* OR Aboriginal* or Metis OR Inuit* OR First Nation* OR Native American* OR Native* OR Indian* OR American Indian OR Alaska Native* OR Torres Strait Islander* OR Maori People*	
**AND**	
3. Chronic disease	
• Chronic disease* OR cardiovascular disease* OR CVD OR heart failure OR HF OR stroke* OR heart attack* OR hypertension OR HT OR diabetes OR dementia OR asthma OR cancer* OR COPD or chronic obstructive pulmonary disease* OR heart disease* OR HIV OR AIDS OR depression OR mental health	

### Study selection

Articles were reviewed by two reviewers (SW & SS) over two rounds for study selection. In the first round, titles and abstracts were reviewed according to the eligibility criteria listed below. In the second round, the full text of the included articles were screened to determine if they met the outlined criteria (Table 1).

#### Eligibility criteria

1. Primary intervention involves source of community engagement for design and/or implementation of an intervention
  This study utilized the term intervention in reflection of the medical/healing context, whereby an intervention refers to a treatment (Western or Indigenous medicine), program, tool, source of support or other action taken to treat or improve health outcomes.

2) Distinction of serving an Indigenous population within the study
  We have utilized the World Health Organization (WHO) definition of Indigenous populations to guide this review. The WHO describes Indigenous populations as communities that reside within, or are attached to, geographically distinct traditional or ancestral territories. These communities belong to a distinct cultural group, descended from the communities present in the area before colonization was introduced and modern geographical borders were defined. Indigenous communities generally maintain cultural and social identities, as social, economic, cultural and political institutions, separate from the mainstream or dominant society or culture. For this review, we did not limit our study selection to specific Indigenous populations or modern states such as Canada or Australia, but instead considered the eligibility criteria met if any of the various Indigenous-relating terms were used define a study population (Table 1).
3) Study population has at least one chronic disease related condition (i.e. heart failure, diabetes, etc.)
  This study uses the Center for Disease Control and Prevention (CDC) definition of chronic disease to guide the study selection process. The CDC describes chronic disease broadly as a condition that lasts 1 year or longer that cannot be prevented by vaccines or cured by medication. Chronic diseases require ongoing medical attention and tend to limit daily living activities. The most common chronic diseases include heart disease, stroke, cancer, chronic respiratory diseases and diabetes. For this review, we have not limited the studies to the most common chronic diseases, as communities often have varying prevalence in specific chronic diseases depending on their geographical location and contributing SDH.

Studies that focused on describing modes to conduct Indigenous research but did not include the implementation of any of its aforementioned suggestions, within a current or proposed study, were excluded. The rationale for this exclusion criteria mainly was due to the vast amount of literature available regarding Indigenous research methodologies and ways of mutual learning. It has been found that a large majority of Indigenous research continues to explore approaches to build partnerships between academics and communities, due to the history of ineffective and temporary pilot projects that have been conducted [1]. Despite the significance of this research, the focus of this review was to evaluate how these recommendations for Indigenous community engagement have been utilized within present studies, and if they have met the minimal standards outlined by previous Indigenous-based PR.

### Charting and extracting data

Studies meeting the inclusion criteria were critically reviewed (SW & SS) using Arksey and O’Malley’s descriptive- analytical narrative method [14]. A data extraction form was developed by the one reviewer to obtain relevant study information This included the year of publication, study location, chronic disease, study design, intervention type (i.e. mobile health tool, workshop, program, etc.), methodology and level of community engagement.

### Summarizing and reporting results

A numerical analysis of the extent and nature of the studies was conducted using tables and chart mappings. To evaluate the extent community engagement strategies were incorporated, this review assessed the presence of community engagement across the various components of the research process. With the varying types of study designs and interventions being developed, we expect that the level of engagement will differ to meet the varying expectation of the studies aims. However, with this, if previous work had been completed in reflection of any partnership or community engagement efforts, this was also taken into account when evaluating the studies level of engagement.

The descriptive data was analyzed using conventional content analysis to conduct a narrative synthesis [14]. In accordance with general PR principles for community engagement, two reviewers (SW and SS) examined the descriptive data and identified key codes relating to the research question [14, 16]. These codes were then organized into thematic groups to summarize the studies according to their main findings.

## Results

**Fig. 1:**
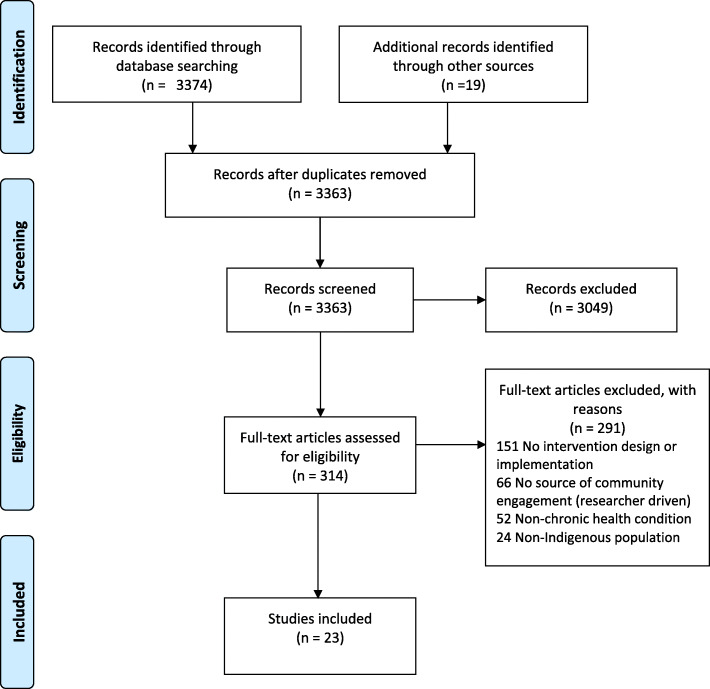
Preferred Reporting Items for Systematic Reviews and Meta-Analyses (PRISMA) flow diagram - Systematic Scoping Review Search Strategy and Study Selection

A total of 3393 articles were identified from the five databases and reference lists searched. Thirty duplicate articles were removed, and the remaining 3363 articles were screened according to the eligibility criteria listed above. After a review of the title and abstracts, only 314 articles were included for the full-text review. Following the second round of review, 293 articles were excluded due to a series of implications with the eligibility criteria, as highlighted in Fig. 1. In total, 23 articles were included in this systematic scoping review (Fig. 1).

### Study characteristics

**Fig. 2:**
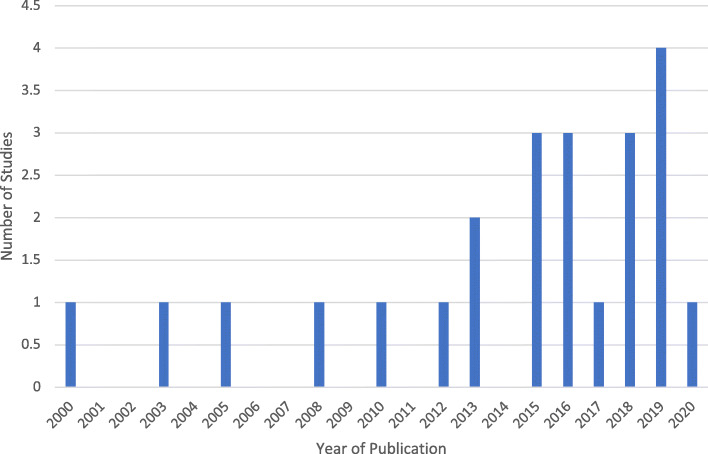
Frequency of Scoping Review Articles According

From the 23 studies, the majority (*n* = 21) were all published after 2010 (Fig. 2). This finding was expected, as it was only in 2007 that the United Nations Declaration on the Rights of Indigenous Peoples (UNDRIP) was adopted by the General Assembly [17]. The UNDRIP initiative essentially sparked the need for various countries to not only recognize Indigenous health disparities, but to also respond with an appropriate mode of action. Australia, Canada, United States (US) and New Zealand were originally the four countries against UNDRIP; however, they have since reversed their positions and expressed support for the Declaration either through a formal or informal endorsement [17]. Originally, they justified their lack of support for UNDRIP as they claimed that Indigenous rights were already being recognized within their own national governance systems. In 2010, Australia, New Zealand and the US announced their decision to support UNDRIP in order to continue to uphold their commitment to supporting Indigenous rights. Conversely, while Canada supported the “spirit” of the Declaration in 2010, it was not until the change to a Liberal government, under Prime Minister Justin Trudeau in late 2015, that Canada officially adopted and promised to support UNDRIP. As a result of these events, this scoping review coincidently also found that the most common study locations were Australia (*n* = 12), followed by Canada (*n* = 6), United States (US) (*n* = 3) New Zealand (n = 1) and one multi-country study composed of Australia, Canada and the US (n = 1) (Table 2).

**Table 2:** Charting of Scoping Review Studies

Author, Year of Publication	Study Location & Population	Chronic Disease	Study Design	Intervention	Methods	Level of Community Engagement
Boyer et al. 2005 [18]	USA- Alaska Natives	Obesity, diabetes and CVD	Cross-sectional study to investigate interactions between genetic & environmental risk factors contributing to excess body weight, diabetes and CVD	Program for chronic disease and healthy behaviour education	1. Collect health and wellness data through interviews and self-administered questionnaires 2. Collect blood samples to test Cholestatic lipid levels and HbA1 levels for long-term glucose maintenance 3. All results were explained to participants when they checked out	Used participatory research model to engage with Alaskan Native villages. Follows model where researcher generates question based on evidence and then goes to community to evaluate if individuals share same goals as researchers. Specifically, met with local tribal councils to obtain study approval. Hired local bilingual (English and Yup’ik) research assistant to explain research to community members and conduct the study. Uses community leadership to develop capacity to address health issues. Created formative evaluative process where two external evaluators monitor study and prepare reports on progress to distribute to village.
Bradford et al*,* 2015 [19]	Australia- Indigenous Australians	CVD	Qualitative co-design study to customize cardiac program for Indigenous People	Cardiac rehab program with: 1. Home care with clinical portal and mentoring 2. Education via videos and motivational messaging 3. Smartphone app with health diary and education	1. Customized three main components of program (service delivery, education, app) by conducting workshops and interviews with healthcare providers	CSIRO met with mayor and council of remote Aboriginal community and obtained local letter of support to conduct study. Team actively engaged with six relevant healthcare providers who specialized in cardiac and/or Indigenous health, as well as an Indigenous production company to adapt program. Provider interviews led to changes in service delivery of program and educational material (i.e. inclusion of mentors, culturally appropriate pop-up messages). Production company re-designed mobile app with Indigenous artwork and graphics to improve cultural appropriateness. Community members were not consulted to evaluate adapted program.
Chambers et al. 2015 [20]	USA	Diabetes	Feasibility study evaluating use of a family-based, home-visiting diabetes prevention/ management intervention for AI youth with or at risk for type 2 diabetes	Diabetes prevention/ management program with home-based lifestyle education and psychosocial support, facilitated referrals, and community-based healthy living activities	1. 9-month community needs assessment with advisory boards and steering committee of tribal, regional and national content experts. 21 round table discussions and 29 interviews conducted with AI youth 2. 12-month intervention phase (single group pre-post). Demographic, participant satisfaction and measures related to knowledge, behavioural and psychosocial outcomes collected 3. 6-month maintenance phase with monthly visits with youth and their support person	Indicated utilizes CBPR approach for design, implementation and evaluation of the study. Both stakeholders and AI youth were consulted for design of intervention features but were not consulted post-design. TOD program utilized 30-year trusted relationship with tribal communities to initiate intervention evaluation phase. During 12-month trial, youth were provided with home-based education on healthy living goals and diabetes prevention. Following the trial, monthly visits with AI youth were conducted to help sustain behaviors. Study claimed it would include participant satisfaction questionnaire to engage AI opinion, but no results are reported.
Ciccone et al. 2019 [21]	Australia- Aboriginal Australians	Stroke	Pilot study using single case ABA design to test feasibility and acceptability of a rehabilitation program for Aboriginals with acquired communication disorders post-stroke	Home-based rehabilitation program with therapy sessions with Aboriginal care worker	1. Eight Aboriginal participants were provided 24 treatment sessions over 12 weeks by a SLP and Aboriginal co-worker 2. Feasibility measured through the number of sessions conducted and participant attendance 3. Acceptability measured through participant post-therapy questionnaire and SLP semi-structured interview	Study utilized Aboriginal framework for research based on their previous Missing Voices study that involved extensive consultation with the Aboriginal Controlled Community Health Organization. Participants were asked to verbally provide their opinion on the key components of the therapy and to rate them on a 3-point scale. “Yarning” style interview was used, form of storytelling, to help improve dialogue between SLP and Aboriginal patient. As a result, participants indicated that having therapy at home created a better environment to obtain knowledge and continue discussions.
Clark et al.*,* 2015 [22]	Australia- Aboriginal and Torres Strait Islander	Heart Failure	Mixed-methods study to re-design existing HF educational resource to be culturally safe	Fluid-Watchers- Pacific Rim HF education resource on an electronic tablet or computer	1. Phase 1 used action research methods to develop a culturally safe electronic resource to be provided to Aboriginal HF patients via a tablet computer 2. In Phase 2, the new resource was tested on a sample of Aboriginal HF patients to assess feasibility and acceptability. Patient knowledge, satisfaction and self-care behaviours were measured using a pre-post design with validated questionnaires.	Utilized various participatory methods in Phase 1 and 2 to adapt and evaluate program. In Phase 1, an HF expert panel adapted the existing resource to ensure it was evidence-based and contained appropriate images that reflect their culture. A stakeholder group composed of Aboriginal workers, HF patients, researchers and clinicians, then reviewed the resources, and changes were made accordingly (i.e. appropriate skin tone/voice, plain language). In Phase 2, Aboriginal HF patients were consulted to evaluate their satisfaction and self-care abilities with the tool.
Crengle et al*,* 2018 [23]	Australia, Canada, New Zealand	CVD	Cross-country, multisite pre–post trial to assess the effect of a customized, medication health literacy program on medication knowledge	Structured CVD health literacy program with: 1. Nurse led education session 2. Interactive tablet and information booklet	1. Three education sessions delivered over 1 month. Medication knowledge and health literacy skills were assessed before and after each session (pre-post).	Study indicates the principal investigators in all three countries are Indigenous and all the researchers have pre-existing relationships with Indigenous communities. Communities were involved in all aspects of project including study design and implementation. Intervention was delivered by registered nurses who had received training in health literacy and adult education. An interactive tablet application was used during each session, which also produced a customised pill card for each participant. Information in the tablet and booklet was standardised across all countries, but background graphic design, images and Indigenous language words were country specific. Study categorized all Indigenous populations as facing similar risk factors and patterns of health inequities, did not consult patients post-session regarding their perspective on the intervention.
Davidson et al*,* 2008 [24]	Australia- Aboriginal Australians	CVD	Mixed-methods prospective study assessing feasibility and acceptability of a collaborative model of cardiovascular education for Aboriginal Health Workers (AHWs)	Education course regarding CVD care for AHWs with: 1. Group education course 2. Clinical education visits	1. Participants were required to complete two questionnaires developed by steering committee assessing knowledge and confidence with CVD pre-post course 2. Semi-structured interviews were conducted 1-month post course completion with all participants	The core course curriculum was based on the AHWs Heart Health Manual. Principles of action learning guided the sessions conduct, where AHWs worked together to complete assessments. AHWs were also encouraged to relate information to their workplace and develop plans to be enacted upon their return. Two questionnaires evaluated their CVD knowledge and confidence levels with respect to CVD knowledge, skills, communication. At the end of the study, participants were interviewed about their perceptions regarding the relevance of course content, needs, use of and barriers to the use of course information, course impact on practice, and interest in further training.
Dimer et al*,* 2013 [25]	Australia – Aboriginal Australians	CVD	Mixed-methods study evaluating impact and uptake of a cardiac rehabilitation (rehab) program	Cardiac rehab program: 1. Education sessions on CVD 2. Exercise sessions	1. Consultation phase- Focus groups with Aboriginal health professionals and community members 2. Implementation phase- Cardiac rehabilitation program conducted once a week with core features for CVD management, education and exercise retention 3. Evaluation phase- Interviews, questionnaires, yarning sessions as well as objective assessment of cardiovascular risk factors. Changes in risk factors were evaluated pre- and post- program using paired t-tests	Study used focus groups in first phase to ensure the program met Aboriginal community needs and expectations. The cardiac program was established under the auspices of Derbarl Yerrigan Health Service (DYHS) (a community controlled AMS) and conducted onsite to provide a culturally secure environment for the provision of exercise and education to address cardiovascular health. Education sessions employed using process of ‘yarning’, which is important in Aboriginal culture for transferring knowledge, building trust and establishing relationships. Visual models were used to illustrate educational messages and reinforced with experiential learning opportunities. Culturally appropriate merchandise (shirts), educational and health promotion resources (including fridge magnets, wallet cards) were produced to support the program. Program became community meeting place, creating an environment of support.
Farmer et al. 2016 [26]	New Zealand- Māori Peoples	Diabetes	Participatory study to develop a culturally relevant diabetes prevention documentary	Educational documentary for diabetes prevention	1. Partnership development- Obtained local support and established community advisory board (CAB) 2. Key informant interviews- Recruit health workers with direct experience working with Māori clients with or at risk for type 2 diabetes 3. Hui (focus groups)- Four hui groups conducted to generate action-oriented of community perspective on diabetes 4. Documentary production- Video was designed and edited with feedback from CAB and group of six Maori women	Principles of CBPR were applied to qualitative research design. First gained the support of local Elders. CAB was created to give researchers guidance on the research design to ensure cultural relevance was maintained and cultural protocols were observed. CAB also helped to interpret the research and analyze the data. Interviews helped to develop understanding of Māori beliefs about diabetes and barriers and facilitators to physical activity and healthy eating. Hui’s led by members of CAB and at end of session participants were asked to provide additional feedback. Hui functioned as a social occasion to discuss family and spiritual aspects of well-being. Documentary purpose/story was initiated by CAB and Maori women revised content to ensure it was culturally appropriate.
Haynes et al*,* 2019 [27]	Australia- Aboriginal Australians	Heart Disease	Co-design, implement and evaluate the rheumatic heart disease (RHD) prevention program in a remote Aboriginal community	RHD prevention program – educational course	1. Activities related to understanding and addressing RHD social determinants were delivered through an accredited course adapted to meet learner and project needs 2. Data collection comprised of focus groups, interviews, observation, and co-development and use of measurement tools. 3. Data analysis utilised process indicators from national guidelines for Aboriginal research	Utilized CBPR research design throughout various phases of study. Community leader identified RHD as issue of priority and worked with researchers to design and implement program. Community members were invited to participate in the study and collaborate on adapting the course for community needs. Study indicates that the lessons learned from this study will be used in next stages of the RHD elimination strategy. This includes strategies to scale-up community leadership in research agenda-setting and implementation.
Kakekagumi-ck et al*,* 2013 [28]	Canada- Sandy Lake	Diabetes	Mixed methods study evaluating prevalence of diabetes in Sandy Lake and obtaining population data to develop culturally appropriate intervention to improve diabetes management (i.e. improve diet and increase physical activity)	1. Northern Store program to increase availability of healthy food 2. Home visit program 3. Diabetes radio show 4. Diabetes curriculum for grades 3 & 4 5. Community-wide walking trail to increase physical activity 6. Diabetes summer camps	1. Formative qualitative/ ethnographic study (1991–1996): Collected information on health beliefs and perceptions of food and physical activity, determinants of health 2. Community surveys (1993–1995): Documented T2DM prevalence and risk factors 3. Used ethnographic data and survey data to develop culturally appropriate intervention strategies	From 1991 to present, they have used formative methods, feasibility research and pilot testing to identify strategies to improve healthy eating and physical activity. The program has received community support, including participation from the community and partnerships with other programs and organizations in the community. All programs are culturally appropriate and have been adapted to suit the needs of the community through a participatory research approach. Over the 22 years of existence, the community has taken ownership of the program and activities have evolved in alignment with community needs and priorities. This program has been refined in an iterative fashion, with reciprocal capacity building for both key community stakeholders and academic partners.
Kholghi et al. 2018 [13]	Canada – Quebec- Kanien’kehá:ka (Mohawk)	Diabetes	Sequential mixed-methods study evaluating Health Education Program (HEP) for diabetes prevention amongst Indigenous children	HEP for diabetes prevention with group sessions on knowledge and skill development for healthy eating and physical activity	1. Cross-sectional survey for 23 teachers 2. Interviews of two elementary school principals 3. Three culturally appropriate Indigenous talking circles with HEP authors, teachers and parents	Study was guided by a participatory research approach that engaged the researchers and the Kahnawake Schools Diabetes Prevention Project Community Advisory Board in shared decision making throughout the project. Talking circles used to discuss the specified topic, this method is a more culturally appropriate increasingly being used in research by Indigenous researchers. Identified barriers such as need for cultural content and outdated resources, which will be used to refine program. This study did not consult with students to gain their perspective.
Peake et al.*,* 2020 [29]	Australia - Aboriginal Australians	Stroke	PAR study that engaged with Australian Aboriginal communities to develop culturally appropriate stroke health resources	Stroke education booklet	1. Community engagement- Collaborative yarning session with Aboriginal Elders followed by series of working groups with members of the community 2. Evaluation- Researchers analyzed engagement session recordings to develop stroke education resource. Previous participants consulted in cycles to confirm design of resource.	Utilizes two stages of PAR to design stroke resource. In phase 1, they developed relationships with various members of the Aboriginal community. Collaborative yarning was used to share stories and identify needs for stroke booklet. Photographs and stories from the Elder were incorporated within the book to encompass simple connection to physical and spiritual environment. Group discussions were led by community members. In phase 2, researchers co-designed tool by creating drafts of booklet and obtaining feedback from Aboriginal organizations and community members. Signed support letters were obtained, and researchers continue to maintain contact with community through monthly visits.
Peiris et al*,* 2019 [30]	Australia - Aboriginal Australians	Chronic Disease	Mixed-methods study aimed to develop and evaluate the feasibility of the electronic platform for community-based chronic disease screening for Aboriginal people	1 Deadly Step Program with: 1. iPad screening app 2. Provider portal and web-based reporting tool 3. Education events	1. Program enhancements: iPad screening app, administrator portal, provider portal and web-based reporting tool added to 1 Deadly step program 2. Evaluation: Mixed-methods evaluation comprising of risk factor analysis, survey data, and analytics from iPad and Web portal. Detailed interviews with key health service and program staff also conducted.	The 1 Deadly Step program was previously developed in partnership with New South Wales (NSW) Health and the Australian Rugby League to address the high prevalence of chronic diseases in NSW Aboriginal communities. Used a culturally safe, innovative, community-based model where annual events are held to increase awareness of chronic diseases and to promote prevention, early detection, and management. Uses popularity of rugby league in Aboriginal communities to encourage local communities to participate. Developed electronic platform according to previous feedback regarding program needs. App was developed with Aboriginal local artist. Program data, participant satisfaction surveys and staff interviews were used to evaluate feasibility.
Quinn et al. 2017 [31]	Australia- Aboriginal Australians	Chronic Disease	Mixed-methods study aimed to re-design and evaluate impact of Get Healthy Service (GHS) for healthy behaviours in Aboriginal communities	Program for healthy behaviours with: 1. Phone-based education and health tracker 2. Cultural referral pathway	1. Interviews with Aboriginal participants, leaders and community members, healthcare professionals and service providers for program re-design 2. Interview and questionnaire to evaluate design appropriateness 3.Quantitative pre-post evaluation of anthropometric measures, physical activity and fruit and vegetable consumption of Aboriginal participants	The GHS is a phone-based service supporting adults to make sustained improvements in healthy eating, physical activity, reducing alcohol intake and achieving or maintaining a healthy weight. Study interviewed Aboriginal participants, leaders and community members, healthcare to examine acceptability of the GHS and redesign GHS for Aboriginal context. Re-design was further confirmed with follow-up appropriateness study. Aboriginal participants recruited for pre-post study. Positive results used to educate communities and build awareness for future use of GHS.
Rowley et al*,* 2000 [32]	Australia- Aboriginal Australians	Obesity, Diabetes, CVD	Cohort study assessing the sustainability of the Looma Healthy lifestyle program for prevention of chronic disease	Healthy lifestyle program with: 1. Cooking classes 2. Health promotion events 3. Walking groups	1. 6-month intervention interval with total of 49 participants over 24 months. Evaluated impact of intervention on body mass index, glucose tolerance, and plasma insulin and triglyceride concentrations	Previously consulted community to understand chronic disease risk factors. Several Aboriginal Health Workers were employed to run program operations. Following study, researchers worked with community to develop policies to enable community control and ownership of program to ensure key changes can be made in accordance with their risk factors of priority.
Shepherd et al*,* 2003 [33]	Australia - Aboriginal Australians	CVD	Quantitative needs assessment to identify barriers to Indigenous patients taking up the cardiac rehabilitation program	Cardiac rehab program with: 1. In-service education 2. Cardiac therapy by Indigenous health worker	1. Cross-sectional survey of stakeholder knowledge (Indigenous cardiac patients and health professionals) and views	Established a steering committee, all members of which lived and worked in the communities targeted, to guide the project. A cross-sectional survey of stakeholder knowledge and views in regard to cardiac rehabilitation was designed. The survey took place in six communities, which are part of the North Queensland Rural Division of General Practice. Stakeholder feedback used to create themes regarding barriers to rehab program. Themes were not followed up or confirmed with participants.
Smylie et al*,* 2018 [34]	Canada- First Nations	CVD	Pre-post study to test the effect of a customized, structured health literacy educational program for CVD medications	CVD educational program with: 1. Nurse led education session 2. Interactive tablet and information booklet	1. Three education sessions delivered over 1 month. An interactive tablet application was used during each session and an information booklet and pill card was provided to participants. Medication knowledge and health literacy skills assessed before and after each session (pre-post).	Applied an Indigenous community participatory action research partnership method that had been successfully demonstrated in a previous community-partnered, community-implemented health needs assessment project. The research team was led by Indigenous people. There was a local project research committee comprised of DHAC staff. Indigenous governance and management of research data and publications was formalized through a signed research, data-sharing and publication agreement.
Tibby et al*,* 2010 [35]	Austrailia – Indigenous Australians	CVD	Mixed-methods study to improve access to specialist cardiac care in rural and remote areas across Queensland.	Outreach specialist cardiac care: 1. Cardiac team (cardiologist, sonographic, Indigenous care workers) 2. Portable echocardiograph machine, ECG, point of care technology and vital signs monitor. Each patient receives an ECG, point of care testing, ECHO and blood analysis	Phase 1: Engagement - Approached Indigenous Care Workers to evaluate their current role and assess if further cardiac training required - Created initial cardiac screening tool in consultation with community to assist direct referral to the outreach program Phase 2: Recovery Intervention - Cardiac service team with a portable echo machine traveled to each site to assess patients and determine care plan Phase 3: Capacity Building - Program coordinator has devised a plan to encourage selected community members to be trained to provide framed programs to improve recovery after cardiac events as well as to improve primary cardiac health	Researchers had pre-established relationship with Indigenous communities in Queensland. Each of the communities were able to articulate their problems and concerns, as well as could voice what they needed for their community before start of study. Researchers established partnership and gave 10-year plan for project completion. Phase 1 involved series of community engagement sessions with Indigenous health workers to understand their role and how to provide ‘care that resonated with historical traditions and intrinsic values of the communities. A cardiac assessment tool was devised, in consultation with the communities, to enhance the relationship and ownership of the Program at the local level. However, patients were not consulted about their cardiac needs or challenges. Phase 2, cardiac team travelled to each community and specialist also provided patient care letter Indigenous Health workers when returning to tertiary referral centre as mode of improvement. Researchers indicate that Phase 3 is in process.
Tobe et al*,* 2019 [36]	Canada- First Nations	Hyper tension (HT)	Multicenter randomized control study to assess the effect of active or passive intervention on blood pressure (BP) levels	HT management intervention with: a) active: HT specific SMS messages b) passive: general health behaviors SMS alone	1. Participants receive either active and passive, or only passive SMS intervention. Passive SMS described healthy lifestyle and behavior changes. Active messages included information on the management of hypertension as well as advice to follow-up with the participant’s health care provider if the measured BP was above target. Individual BP measurements were taken by community health workers using an automated BP device with Bluetooth transmission capability.	Utilized PAR research approach for design, conduct and evaluation of the study. In previous studies, built partnership with Indigenous communities, where key stakeholders involved in designing study tools and evaluating data. All SMS text messages were derived from the Hypertension Canada Clinical Practice Guidelines and modified with community input to make them culturally sensitive. The study team and researchers included expertise appropriate to the intervention including clinical trials with Indigenous communities, hypertension measurement and management, program management and technical knowledge, participatory health research, Indigenous community health and research ethics, consequences of colonization and the Indian Residential Schools on Indigenous health, clinical trials design and statistics
Wakewich et al*,* 2016 [37]	Canada- First Nations	Cervical Cancer	Multi-community qualitative study investigating stakeholder perception on the cervical cancer screening initiative	Cancer screening program	1. Conducted 16 interviews with healthcare professionals 2. 9 focus groups with 69 women from the communities	Study is part of a larger public health initiative with First Nations women in Northwestern Ontario to develop culturally sensitive approaches to cervical cancer screening. Argue that education and screening initiatives must reflect the cultural preferences of Indigenous women, empowering them to take control of their experiences of health and body in cervical cancer screening. Principal investigator is a non-Indigenous cancer biologist who has worked with First Nations leaders and health representatives from 10 communities in Northwestern Ontario to define project priorities and optimal ways to actively engage community members. A PAR framework aimed at fostering collaboration between HCPs, community members and academic partners, has guided all stages of the study. Also conducted educational workshop to share findings and obtain feedback from the study participants. Discussions and meetings were continued during visits to the partner communities.
Walters et al*,* 2012 [38]	USA- Alaska Natives	CVD	Randomized control trial to 1) to assess feasibility and initial efficacy of a culturally adapted intervention targeting diet and exercise compared to a family life-skills building intervention and 2) to strengthen tribal research infrastructure	a) CVD prevention intervention with focus on diet and exercise b) Family life-skills building intervention with focus on cohesiveness and connectedness	1. At-risk adults (*n* = 135) were randomly assigned to either a CVD prevention intervention arm or a comparison arm focusing on increasing family cohesiveness, communication, and connectedness. Both year-long conditions included 1 month of motivational interviewing counseling followed by personal coach contacts and family life-skills classes. 2. Blood chemistry, blood pressure, body mass index, food intake, and physical activity were measured at baseline and at 4- and 12-month follow-up	This project was a multidisciplinary collaboration among experienced tribal care providers. All parties were committed to a CBPR approach, which included respect for the tribes’ autonomy, sovereignty, and confidentiality, as well as subsequent dissemination of the findings to further the implementation of culturally relevant and cost-effective programs. Interventions were designed to incorporate cultural components and to emphasize a holistic approach to health consistent with Indigenous values. Last phase of the project involves results dissemination to the tribal council, community, and health care providers. Plan to hold at least two community town meetings to share the findings from all phases of the research project and to elicit feedback on data interpretation, risks and benefits to community, dissemination strategies, community readiness to implement intervention and transferability to other tribal nations.
Ziabakhsh et al*,* 2016 [39]	Canada- Indigenous women	CVD	Pilot study evaluating Seven Sisters pilot intervention on participant health outcomes and the processes that contributed to them (how participants perceived the intervention)	Heart healthy program with: 1. Weekly talking circles for education	1. Demographic and health profile data were obtained with an intake form 2. Eight two-hour, weekly women only talking sessions to discuss various CVD-related content and Indigenous health practices. Participants also completed a questionnaire in the first and last sessions that included questions on diet, physical activity, and smoking	Seven Sisters project was piloted as a gender- and culturally responsive model to promote heart-healthy activities among indigenous women. Project builds on previous research by using traditional processes and traditions (e.g., talking Circle) but also integrates Indigenous knowledge into mainstream health promotion messages and practices and promotes a holistic approach to heart health among women. A participatory and a developmental approach to evaluation was taken, as the program evolved, the focus of the evaluation also changed. Participants were given opportunity to comment on all aspects of the program, and researchers aim to modify program according to their suggestions. Data was analyzed by study team and cultural lead for triangulation and validity check.

With respect to the chronic disease of focus, the majority of the studies specifically targeted cardiovascular disease (CVD) (*n* = 9), diabetes (*n* = 4) or combined various chronic diseases within its intervention (i.e. CVD, diabetes, obesity) (n = 4) (Table 3). The intervention’s used within the various studies were mainly for 1) Education only (chronic disease information, medication literacy and healthy lifestyle) (*n* = 17), 2) Education + home care (clinical visit or rehabilitation) (n = 4), 3) Home care only (clinical visit/rehabilitation) (n = 1) or 4) Disease management (*n* = 2). Nevertheless, despite the intended use of the interventions, there were varying features included to enhance its impact on Indigenous health outcomes. The most common feature or component included was the use of a mobile phone or tablet (*n* = 7). Indigenous populations residing in more geographically remote areas are at an even further disadvantage and a higher burden of chronic disease, thus the use of a mobile phone allowed them to obtain their needed support more readily [2]. Other intervention features included the use of visual aids (*n* = 4), community referrals (*n* = 3) and exercise sessions (n = 3) (Table 4).

**Table 3:** Summary of Scoping Review Chronic Diseases of Interest

Chronic Disease of Focus	Articles ***n*** (%)
Cardiovascular disease (CVD)	9 (39)
Diabetes	4 (17)
Combination of chronic diseases (CVD, diabetes, obesity)	4 (17)
Stroke	2 (9)
Heart Failure	2 (9)
Hypertension	1 (4)
Cancer	1 (4)

**Table 4:** Breakdown of Intervention Features in Scoping Review Articles

Intervention Feature	Articles ***n*** (%)
• Mobile phone or tablet app (health diary, education, disease management/reminders)	7 (30)
• Videos, motivational messaging, visual models within tools	4 (17)
• Community referral system	3 (13)
• Exercise sessions	3 (13)
• Portable clinical devices	1 (4)
• Cooking classes	1 (4)
• Clinical portal and mentoring	1 (4)
• Program merchandise (shirts, fridge magnets, wallet cards for education)	1 (4)

### Level of community engagement in research process

With the varying types of study designs and interventions being developed, we found that the level of engagement used across the 10 components of the research process differed to meet the varying expectation of the studies aims (Table 5). As a result, despite the majority of studies indicating the use of a specific PR model or framework (*n* = 18), only 6 studies *involved* community members to confirm the area of priority, whereas only 1 actually had the community identify the area of priority themselves (Table 5). Conversely, the most common level of engagement shared by the studies were at the research approval (*n* = 11) and intervention modification (*n* = 11) phases. Research approval generally involved sources of Elder, community committees or tribal council support. To further understand the level of engagement used for intervention modification, this review subdivided this section in accordance with the extent various different community members were involved. Specifically, the level of engagement differed by interviewing or obtaining feedback from 1) community members + stakeholder (i.e. Elder, healthcare provider) (n = 11), 2) healthcare providers only (*n* = 5) and 3) community members only (*n* = 3). However, from these studies it is important to recognize that only four actually engaged with community members and/or stakeholders both before and after the intervention’s design, to confirm if feedback was appropriately interpreted and accommodated for.

**Table 5:** Level of Community Engagement Used in Each Phase of Research Process

Study Component	Example Study Activity	Articles ***n*** (%)
Initiation of Partnership	• Researchers from University of Canterbury invited by Māori Peoples in New Zealand to discuss partnership and potential study goals and priorities [26]	7 (30)
Research Approval	• Australian research group met with mayor and council of remote Aboriginal community to obtain local letter of support to conduct study [19]	11 (47)
Research Question, Study Aims	• Community members and Elders involved in discussion that determined study goals [32]	6 (26)
	• Community members and Elders identified and determined study goals [27]	1 (4)
Design, Methods, Approach	• Community advisory board was created to give researchers guidance on the research design to ensure cultural relevance was maintained and cultural protocols were observed [26]	2 (9)
Protocols	• Local research assistant hired to co-develop surveys and interview guides with research team [18]	2 (9)
Data Collection	• Members of Indigenous community involves in both recruitment and moderation focus groups and took notes [34]	3 (13)
Data Analysis	• Community members involved throughout data analysis process by coding and reviewing themes [23]	5 (22)
Intervention	• Stakeholder group composed of Aboriginal workers, HF patients, researchers and clinicians reviewed electronic resource, then changes were made according to feedback (i.e. appropriate skin tone/voice, plain language) [22]	11 (47)
Dissemination	• Created formative evaluative process where two external evaluators monitor study and prepare reports on progress to distribute to village [18]	3 (13)
Sustainability	• Used community leadership to develop local capacity to sustain program [28]	6 (26)

### Findings from narrative synthesis

Two dominant themes related to the different levels of community engagement used within the included studies were identified: 1) Adapting for the local cultural context and 2) Inclusion of community outreach. The first theme characterizes the various modes in which studies have either used recommended Indigenous methods or have incorporated community feedback within the interventions design. The second theme mainly entails the efforts researchers have made to involve and inform community stakeholders both within the research process and after the study has ended (Table 6).

**Table 6:** Summary of Community Engagement Themes and Corresponding Study Features

Community Engagement Themes			
Adapting for Cultural Appropriateness	Articles ***n*** (%)	Inclusion of Community Outreach	Articles ***n*** (%)
• PR model or Aboriginal framework used	18 (78)	• Met with tribal councils to obtain study approval	11 (48)
• Adapted program features following community feedback (culturally appropriate messages, language, Indigenous art/graphics)	10 (43)	• Used community leadership to develop local capacity to sustain program	6 (26)
• Community members included to analyze/ interpret study findings	5 (22)	• Created partnership or long-standing relationship	7 (30)
• Used yarning style interviews or talking circles	5 (22)	• Researcher identified/confirmed area of priority with community	6 (26)
• Confirmed intervention adaptation following initial engagement	5 (22)	• Created reports on study progress to distribute to communities	3 (13)
• Hired local bilingual research assistant to explain study to community members prior to interview	2 (9)	• Community members led conduct of study	2 (9)
		• Community members identified area of priority	1 (4)

#### Adapting for the local cultural context

Amongst all studies within this review, the importance of adapting for the local cultural context was identified as key component for the uptake and sustained use of an intervention [18–40]. With this, most articles indicated the use of a PR model to ensure the study respected the Indigenous cultural setting (i.e. PAR, CBPR, Indigenous framework) (*n* = 18). However, only 10 studies had actually incorporated the fundamental engagement steps needed within their study approach. This included valuable features such as the inclusion of community members during data analysis (*n* = 5) and the use of Indigenous interview styles to promote meaningful feedback (i.e. yarning or talking circles) (*n* = 4) (Table 6).

In Farmer et al. [26], they conducted a PR study to develop a culturally relevant diabetes prevention documentary, and within their study design they had developed a community advisory board that guided the researchers on all phases of the study. From this approach, they specifically highlighted that allowing community members to interpret and analyze the data was most helpful, as there were significant items the researchers would have missed without their support [26]. Similarly, Ciccone et al. [21] had included yarning style interviews during their pilot study for Aboriginal people with acquired communication disorders post-stroke and found that this method helped improve the dialogue between the provider and Aboriginal patient.

#### Inclusion of community outreach

The majority of the studies’ main source of community outreach involved obtaining study approval from an Elder or local Indigenous ethics council (*n* = 9). Community empowerment was identified as a key feature to support the uptake and longevity of an intervention, but few studies actually included these modes of community outreach. Similarly, it was also found that Indigenous led studies were more widely accepted and had greater potential for sustained growth. However, as previously highlighted, only two studies were led by community members and only a single study actually had the community identify the issue of priority.

Few studies had actually incorporated community outreach items such as developing local capacity for the intervention (*n* = 6) or creating reports to update community members on study progress (*n* = 3) (Table 6). Boyer et al. [18] is an example of a study that actually collaborated with the community to hire a local bilingual (English and Yup’ik) research assistant to help conduct the study. With the support of the research assistant, this study was able to use community leadership to develop local capacity to sustain the program beyond study completion. Boyer et al. [18] had also created a formative process where two external evaluators would monitor the program and prepare reports on its progress to distribute to the village.

## Discussion

### Principal findings

Several studies have highlighted the historical mistrust between Indigenous communities and researchers, that has made the inclusion of PR or community engagement a bare necessity to improve Indigenous health outcomes [41, 42]. However, few have explored or elaborated on the specific process in which modes of community engagement should be initiated to ensure Indigenous cultural values are appropriately respected. In this study, we conducted a scoping review to assess the level of community engagement used when designing or implementing chronic disease-based interventions for Indigenous populations. Across the 23 articles meeting the eligibility criteria, this review found that the majority of studies agreed on the beneficial necessity to engage with community members to ensure the uptake and sustained use of an intervention. These engagement approaches were noted to be able to help allow community members to re-build their connection and sense of belonging with society that had otherwise been neglected within other aspects of the healthcare system.

Despite the agreed benefits community engagement would provide, we found that the studies included in this review utilized varying levels of community engagement or PR methods, which had ultimately limited the overall impact of their work. Studies ranged from involving community members at multiple steps of the research process (i.e. study design, data analysis) to only consulting with the community to gain study approval (Table 5). The lack of a cohesive standard regarding the minimum community engagement efforts to be used to appropriately conduct Indigenous-based research, had ultimately led researchers to conduct studies based on their own interpretation on what level of community engagement was deemed sufficient. Previous studies have developed implementation frameworks to guide researchers in developing and implementing health interventions with Indigenous communities [41–43]. Specifically, the He Pikinga Waiora (HPW) is an example of an implementation framework that has been used in a few of the PR-based studies within this review, to guide their research methodology [42]. HPW is intended to be utilized as a planning tool, whereby Indigenous self-determination is used as the core value of the framework and the four essential elements are used to guide intervention development and/or implementation: 1) culture-centeredness, 2) community engagement, 3) systems thinking and 4) integrated knowledge translation [42]. This framework emphasizes fundamental components in conducting Indigenous-based research including the need to incorporate the community voice, reflexivity amongst researchers, community involvement and shared decision-making to encourage holistic perspectives of health [42]. However, the gap in this framework involves the re-occurring issue in which many frameworks highlight the key principles to be considered when designing or implementing an intervention in Indigenous communities, but they do not specify the specific steps or core requirements to appropriately meet the criteria outlined in the framework. With this, researchers are once again left in the position to make their own judgement on the level of engagement adequate to meet the framework’s guidelines.

With the lack of a clear guideline on conducting PR and the essential criteria to be met for proper community engagement, this review has summarized the supported engagement methods related to the two themes highlighted above, to outline a series of recommendations to guide future Indigenous-based research in this field.

### Recommendations: What’s appropriate vs. What’s feasible

#### Initiation of partnership

With over 80% of studies indicating the use of a PR model or framework, it is anticipated that many researchers may have initially used these approach terms due to its popularity across the literature, without accurately understanding the differences in its engagement levels (i.e. PAR vs CR). To better evaluate the most suitable research approach, we recommend that researchers focus on first initiating a partnership with the community. Ultimately, by initiating a partnership, this would open a dialogue between the community and the researchers, to better evaluate the community’s research goals (Table 7). The process of establishing a partnership also allows for opportunities of mutual learning, whereby various Indigenous knowledges and lived-experiences can be highlighted to facilitate the conduct of a study as well as the design of an intervention. In many cases, the strength behind Indigenous knowledges can be overlooked by Western modes of research, thus the use of these approaches helps to ensure multiple lenses of knowledge are incorporated across the research process [39]. This pivotal step also follows the Tri-Council Policy for Ethical Research Involving Humans (Statement 2, Chapter 9), OCAP and IQ. With the history of Indigenous-settler interactions and power-imbalances, partnership building is an essential component of Indigenous research [39, 40]. By effectively establishing a partnership, not only will this help empower communities to become the champions of their care, but it also prevents researchers from partaking in low priority projects or running into community-based limitations during their study. From this review, we found that only seven articles had initiated a research partnership prior to the start of the study, and coincidently these studies were among the articles that included the most diverse methods of community engagement.

**Table 7:** Summary of Community Engagement Recommendations from Scoping Review Main Findings and Themes

Recommendation	Description of Community Engagement Activities
Initiation of Partnership	• Initiate partnership with Indigenous population through continuous process of trust building and open discussions regarding intentions • Discuss community priorities and potential study to assist with raised issue • Allow community to determine study goals, but researcher should be allowed to probe conversation as necessary (i.e. facilitation not identification) • Researcher should provide details to community regarding various modes of community engagement that can be included to allow community to approve the appropriate methods or suggest alternatives. This includes but is not limited to the following methods: 1) Include community member to oversee or lead conduct of study with researchers 2) Include community member to conduct interviews and/or recruit participants 3) Include community member to conduct data analysis in either English or various Indigenous languages 4) Utilize yarning or sharing circle style interviews (i.e. storytelling) 5) Create reports on study progress to distribute to community
Study Approval	• Following finalization of study methods, obtain final study approval to ensure any overlooked or new issues have not been accounted for
Adaptation of Intervention	• Obtain community feedback both before and after intervention changes to ensure feedback has been appropriately interpreted and accommodated for (i.e. pre and post) • Researchers should evaluate whether inclusion of mobile phone would be beneficial with respect to the outlined intervention goal (i.e. improved access to care, visual aids, motivational messaging)

Within the scope of partnership building, it is important to highlight that researchers should also allow the community to dictate the appropriate level of engagement to be used with respect to the study methods. This feedback may dictate or change the intended study design in regard to whether the community deems the use of PAR, CPBR or CR would be most appropriate. To facilitate discussions regarding the study methods, we recommend that the researchers provide details to the community regarding the various modes of community engagement that can be included (i.e. designing study materials, data analysis, yarning interviews). However, the community should ultimately be given the authority to either approve methods, disapprove or suggest alternatives (Table 7). Indigenous populations around the world face different inequities and have various cultures that may play a role in determining the level and type of engagement methods utilized [41]. Therefore, by giving the community options for the study approach, but still allowing the community to make the determining decision, this allows for the study to move forward in a more integrative and community-driven manner. This approach would also allow the researcher to account for any limitations within the community due to resources available or community principles (i.e. research assistant available, translator, yarning), instead of assuming the inapplicability of a certain method. Once these engagement strategies have been confirmed, this will allow the researcher to determine which PR approach is most suitable. We recognize that the partnership building process often requires a large time commitment. However, it will help to re-establish the trusting relationship that has been lost through years of non-relational research practices, which will ultimately improve the likelihood to bring about meaningful change.

#### Study approval

Throughout the partnership building process, various discussions regarding the study may be informal and not effectively accounted for within the final research protocol. Often times, with the number of steps completed during partnership building, it is assumed that the community has approved the study. Thus, to ensure the discussed methods are reflected in the protocol, it is pivotal to obtain final approval from the community prior to the start of the study and the submission to a research ethics board [36]. We also suggest that as part of the PR and study approval process, multiple community stakeholder meetings are taken on a timely basis (i.e. bi-weekly, monthly, quarterly) to ensure that entire research team is collectively satisfied with the study direction.

#### Adaptation of intervention

Various studies have included approaches in which they obtained feedback from the community (*n* = 19) as a source of initial engagement. However, to ensure community feedback has been appropriately interpreted and accommodated for, we recommend that researchers obtain community feedback both during an initial interview, as well as after the intervention changes have been made. From this review, we specifically found that interventions that added features following community feedback were more widely accepted by its target population, as it gave individuals a sense of connection with the tool (i.e. culturally appropriate messages, language, indigenous art/graphics). For example, in Peake et al. [29] they used a PAR approach to design a stroke education booklet, where community members were involved to both design and confirm the content in the booklet. Feedback from this study emphasized incorporating Indigenous stories within education material, where one participant specifically said, “don’t go sanitizing the stories, we need to tell it how it is”. This participant went on to compare stroke to a blockage in the river, and it was the inclusion of these types of references that connected with the members of the community to promote the uptake of the education booklet [29].

From this review, we also found that a number of key intervention features surrounded the use of a mobile phone (i.e. visual aids, motivational messaging). With the rising use of mobile phones across the world, these interventions can serve as a low-cost platform to support populations with limited access to care [40]. Despite some studies highlighting issues related to poor Internet connectivity that may jeopardize the beneficial impact of mobile phones, the use of offline services (i.e. Internet not required for use) and the general expansion of local broadband coverage in many Indigenous communities has helped to address these technical concerns [31, 36, 40]. With this, we recommend that researchers evaluate the potential applicability of transferring their intervention to be mobile-based or adding a supplemental mobile tool. These tools can be customized and adapted to meet the varying Indigenous community contexts, as well as updated or refined if these needs change. With a mobile tool, individuals have the ability to not only manage their condition but also inform their care provider or healer on any changes in their health. Within our review, Tobe et al. [36] was the only study that had an intervention to support disease management, and this was facilitated through the use of a mobile phone. The use of a mobile phone may not be applicable to all communities or research studies; however, we recommend that researchers consider the benefits of its potential integration.

### Limitations

This scoping review was limited in the number of studies available, due to required criteria of focusing on the design or implementation of an intervention. Many studies were excluded as their focus was to understand the appropriate mode to approach research, or to identify the underlying factors contributing to Indigenous health outcomes such as the SDH or the impact of colonization, both which were not the focus of this review. We also recognize that our search strategy included key terms relating to PR or community engagement. However, studies that did not use these terms or report on their PR efforts may not have been captured due to this limitation.

## Conclusion

The health disparities amongst Indigenous populations remains to be one of the greatest injustices present in society. With the rise in chronic disease prevalence, various interventions have been designed using PR methodologies to better support Indigenous health. Nevertheless, despite the claimed use of PR or community engagement strategies, few studies have adequately used these engagement methods within the design and implementation of their interventions. With the lack of a proper guideline regarding modes to execute PR, we recommend that researchers focus on 1) building partnerships with communities, 2) obtaining study approval and 3) adapting their intervention to fit community needs. Ultimately, there are multiple methods that can be incorporated within a study to improve its effectiveness, but it is important to recognize that as researchers we are facilitating a conversation and it is what the community deems appropriate that is most important.

## Supplementary Information

**Additional file 1: Appendix 1.** MEDLINE Search String.

## Data Availability

All article data extracted for this review can be found in Table 2.

## Abbreviations

CVD: Cardiovascular disease
CBPR: Community-based participatory research
CR: Community research
IQ: Inuit Qaujimajatuqangit
OCAP: Ownership, Control, Access, Possession
PAR: Participatory action research
PR: Participatory research
SDH: Social determinants of health
UNDRIP: United Nations Declaration on the Rights of Indigenous Peoples
US: United States
