# Supplementary material for: What do you mean by engagement? – evaluating the use of community engagement in the design and implementation of chronic disease-based interventions for Indigenous populations – scoping review
Source: Int J Equity Health. 2021 Jan 6;20:8. doi: 10.1186/s12939-020-01346-6 (PMC7788761; doi:10.1186/s12939-020-01346-6)
Supplement: Supplementary file 1 — Additional file 1: Appendix 1. MEDLINE Search String. [file 12939_2020_1346_MOESM1_ESM.docx]

**Appendix 1: MEDLINE Search String**

1. (community based participatory research or community engagement or participatory action research or community institutional relations or community participation or action research).mp. [mp=title, abstract, original title, name of substance word, subject heading word, floating sub-heading word, keyword heading word, organism supplementary concept word, protocol supplementary concept word, rare disease supplementary concept word, unique identifier, synonyms]
2. . (Indigenous or Aboriginal* or Metis or Inuit* or Dene or Gwichin or Athabas* or Inuk or Yup'ik or Inuviat* or Yupik* or Aleut* or Inupia* or First Nation* or Native American* or Upajati, jumma, hillmen, minzu, chuncheat daoem pheak tech, masyarakat adat, Yazidis Assyrian* or Ladakhi or Kinnaur* or Lepcha or Bhutia or Naga or Karbi Bodo or Munda or Mizo or Kodava or Toda or Kurumba or Kota or Irula* or Jat* or Nivkh or Ainu or Dzungar Oirat* or Pamiri* or Ryukyuan or Cham or Degar or Khmer Krom or Javanese or Sundanese or Bantenese or Betawi or Tengger or Osing or Badui or Madurese or Malays or Batak or Minangkabau or Acehnese or Lampung or Kubu or Dayak or Banjar or Makassarese or Buginese or Mandar or Minahasa or Buton or Gorontalo or Toraja or Bajau or Balinese or Sasak or Nuaulu or Manusela or Wemale or Dani or Bauzi or Asmat or Igorot or Lumad or Moro or kurd* or hmong).mp. [mp=title, abstract, original title, name of substance word, subject heading word, floating sub-heading word, keyword heading word, organism supplementary concept word, protocol supplementary concept word, rare disease supplementary concept word, unique identifier, synonyms]
3. (Cardiovascular disease* or CVD or heart failure or HF or stroke* or heart attack* or chronic disease* or hypertension or diabetes or mental health or asthma or copd or cancer* or heart disease or angina or arthritis or bipolar disorder or chronic respiratory disease or depression or multiple sclerosis or osteoporosis or sleep apnea or glaucoma or Alzheimer or Parkinsons or epilepsy or lupus or crohns disease or kidney disease).mp. [mp=title, abstract, original title, name of substance word, subject heading word, floating sub-heading word, keyword heading word, organism supplementary concept word, protocol supplementary concept word, rare disease supplementary concept word, unique identifier, synonyms]
4. . 1 and 2 and 3
